# Calculating Apparent p*K*_a_ Values of Ionizable Lipids in Lipid Nanoparticles

**DOI:** 10.1021/acs.molpharmaceut.4c00426

**Published:** 2024-12-10

**Authors:** Nicholas B. Hamilton, Steve Arns, Mee Shelley, Irene Bechis, John C. Shelley

**Affiliations:** † Schrödinger, Inc., 101 SW Main Street, Suite 1300, Portland Oregon 97204, United States!; ‡ Acuitas Therapeutics, 6190 Agronomy Road, Suite 405, Vancouver, British Columbia, Canada V6T 1Z3; § Schrödinger, GmbH, Glücksteinallee 25, 68163 Mannheim, Germany

**Keywords:** ionizable lipids, lipid nanoparticles, LNP, apparent p*K*_a_, umbrella sampling, molecular dynamics

## Abstract

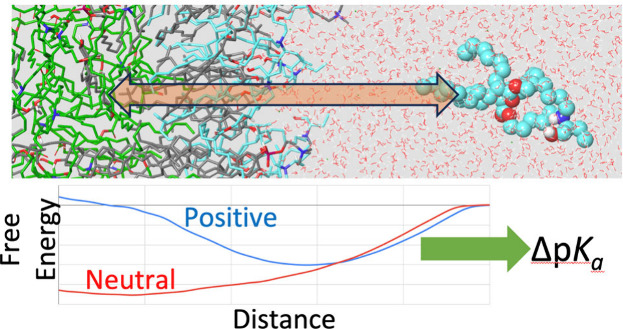

Creating new ionizable lipids for use in lipid nanoparticles (LNPs) is an active field of research. One of the critical properties for selecting suitable ionizable lipids is the apparent p*K*_a_ value of the lipid as formulated in an LNP. We have developed a structure-based, computational methodology for the prediction of the apparent p*K*_a_ value of ionizable lipids within LNPs and have tested it using the lipid formulations in the mRNA LNP COVID-19 vaccines COMIRNATY and Spikevax, and the siRNA LNP therapeutic Onpattro. The calculation was also applied to Lipid A, a variant of the ionizable lipid used in COMIRNATY.

## Introduction and Background

Due to their instrumental role in combating the SARS-CoV-2 pandemic,^1,2^ lipid nanoparticles (LNPs) and the mRNA vaccines they enable are now household names. For instance, as of March 2024, ∼4.6 billion doses of the COMIRNATY (Pfizer/BioNTech) vaccine have been shipped worldwide.^3^ These statistics represent a remarkable achievement for humanity during society’s time of need and these vaccines would not have been possible without the earlier development of LNP delivery systems. As nucleic acid–based therapies, including mRNA, continue to be adapted for new indications and diseases,^4,5^ next generation LNP formulations providing more efficient and selective delivery systems are one way to further enable these therapies.

mRNA is an endogenous messenger molecule that bridges the gap between DNA and the ribosome, enabling the synthesis of protein from instructions in genetic code. The coding architecture of mRNA is readily sequenced^6^ and synthetic methods are well understood.^7^ As such, engineered mRNA sequences can harness the internal mechanisms of the cell to synthesize proteins that have broad therapeutic applications from vaccines to protein replacement to gene editing and more. Despite the elegance of this approach, susceptibility to endogenous nucleases combined with the size and negative charge inherent to long mRNA strands that limits cellular penetration make the efficient delivery of mRNA to the cytoplasm where the ribosome is located a significant challenge.^8^ mRNA encapsulated in an LNP is afforded both robust protection from degradation and a fine-tunable mechanism for optimizing payload delivery.

After uptake into target cells via endocytosis an LNP is exposed to an increasingly acidic environment as the endosome matures. As the pH drops, the amines of the ionizable lipids (ILs) are protonated and the LNP is understood to undergo structural changes as the ILs begin to associate with the anionic endosomal membrane. This process facilitates escape of the encapsulated mRNA from the endosome into the cytoplasm; a crucial step on the path to translation of a protein of interest. It is well-known that the p*K*_a_ values of ILs in bulk aqueous solution (p*K*_a_^S^) are typically much higher than the apparent p*K*_a_ (p*K*_a_^A^) values for the same lipids as measured in LNP formulations.^9,10^ p*K*_a_^A^ values in the specific range of 6–7 is one requirement to observe functional activity with a typical LNP formulation. This p*K*_a_^A^ range allows for particle destabilization and endosomal escape of the mRNA as the endosome acidifies after the LNP is internalized. Additionally, a p*K*_a_^A^ value lower than the pH of the blood (∼7.4) avoids the LNP having significant net positive charge, which is a known driver of LNP toxicity. Finally, this p*K*_a_^A^ range supports effective encapsulation of mRNA at the acidic pH of the formulation process.

Considering these factors, a key design feature in the search for new ILs is having an appropriate p*K*_a_^A^ value when formulated in an LNP.^11−18^ As such, there is considerable interest in developing computational tools to predict p*K*_a_^A^ for LNPs^19^ to support the systematic design of ILs and to increase the success rate of synthesized lipids having ideal LNP p*K*_a_^A^ values. One method for doing so using a coarse-grained model has been recently described in the literature.^20^ This method can take into account structural aspects of the environment, however, some key parameters such as local geometry and an effective dielectric constant are chosen rather than determined. Herein, we present a computational method which can be performed in roughly 1 week and provides reliable p*K*_a_^A^ values for ionizable lipids in LNPs. This methodology utilizes umbrella sampling^21−23^ to quantify the p*K*_*a*_ shift (Δp*K*_a_) of a lipid upon transfer from bulk aqueous solution to an environment that is locally similar to that they would experience in an LNP when not directly associating with a RNA molecule. This shift can in turn be combined with either experimental or computationally derived p*K*_a_^S^ values to yield p*K*_a_^A^ values for specific LNP formulations.

## Methodology

**Figure 1:**
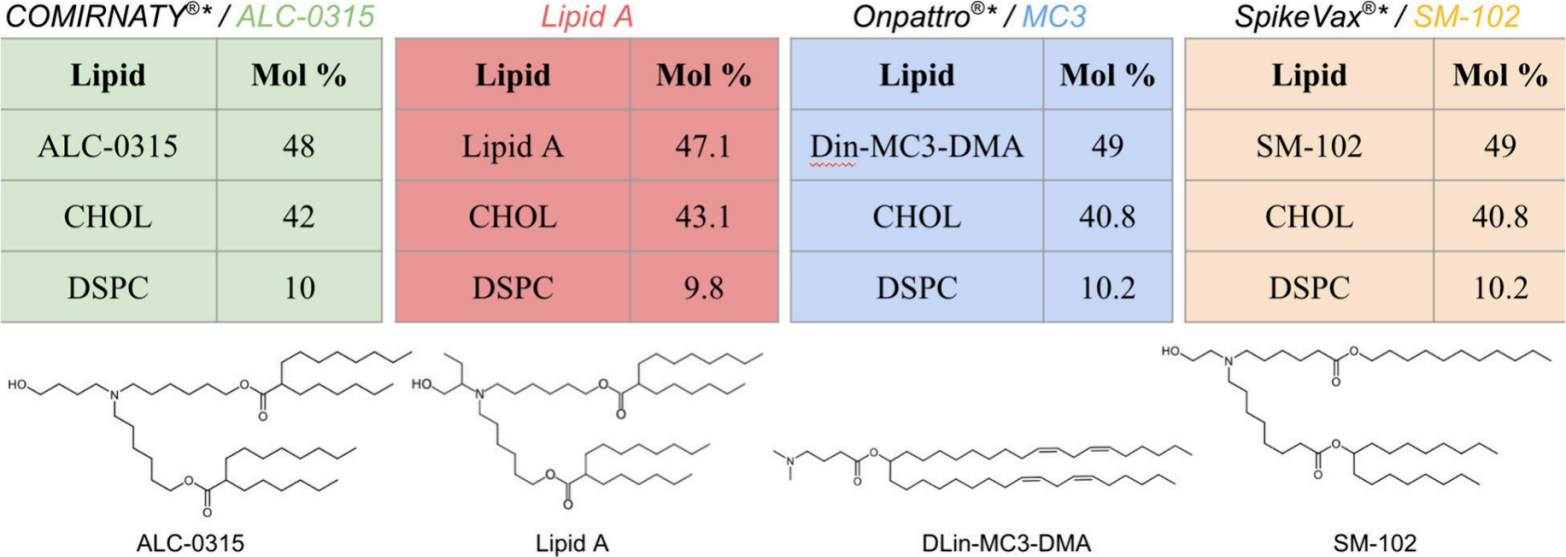
Compositions and lipids used to create LNP bilayers. The four lipid compositions used in this study are based on published compositions of contemporary LNP systems,^3^ omitting the PEG-based lipids, increasing the amounts of the other three lipids proportionally to their mol % values in the full formulation, and rounding off to integers for the actual numbers of lipids. CHOL and DSPC stand for cholesterol and distearoylphosphaticdylcholine. The *’s reflect the absence of mRNA and the PEG-based lipids in our calculations as well as the effect of using an integer number of each lipid in the calculation. The structures for the full ILs for each formulation are also depicted.

The Structured Liquid Builder utility, which is a part of Schrödinger Materials Science Suite^24^ and a front-end to Packmol,^25^ was used to generate the initial lipid bilayers at a surface area per lipid of 60.5 Å^2^. See the Supporting Information (SI) for additional information on constructing these bilayers. Figure 1 gives the compositions for each of the four systems studied (more detailed information is available in Table S1). Two of these systems represent the mRNA LNP formulations used in SARS-CoV-2 vaccines COMIRNATY (Pfizer/BioNTech) and Spikevax (Moderna) while the third represents Onpattro (Alnylam), a siRNA LNP product for treating polyneuropathy of hATTR. Lipid A, a variant of the IL from the COMIRNATY vaccine, ALC-0315, was also studied since it has an unusually low p*K*_a_^A^ value. We leave out the polyethylene glycol (PEG) lipids which comprise approximately 1–2 mol % of the lipids present in these formulations, because the myristol-anchored PEG lipids present in the formulations used in the current study largely shed prior to endocytosis^26−28^ and fine-tuning of the p*K*_a_^A^ value seems to be most sensitive for endosomal escape. mRNA was also omitted from our calculations because including it would require larger systems and likely longer simulations times, and we expect that mRNA will only affect the p*K*_a_^A^ value of ionizable lipids in close proximity (i.e., buried inside the LNP) and evidence suggests that a critical step in the process of endosomal release is for lipids at or near the surface of the LNP to become protonated.^29^ While leaving out these large flexible molecules is an approximation, doing so simplifies the calculations by reducing the required system size and simulation run times. The compositions of the ALC-0315 and Lipid A systems differ slightly because one additional cholesterol molecule was included in each half of the bilayer in the latter due to change in rounding off molecule counts late in the project. The mol % values of the other lipids were proportionately decreased to compensate. The ILs for each of these formulations are depicted in Figure 1. In each system equal amounts of protonated and neutral forms of the IL are included, implying that the pH is effectively equal to the p*K*_a_^A^ for that IL. The built structures included 0.15 M NaCl, as well as additional Cl^–^ counterions to ensure that the system has a net overall charge of zero. While our calculations are based upon a planar bilayer geometry, the interior of an actual LNP may have a range of local water/lipid geometries and this difference may cause shifts of our calculated p*K*_a_^A^ values relative to the experimental values.

Our umbrella sampling approach involves accurately determining the distribution of distances along a straight line, in this case, the distance from the center of membrane to the amine atom in a lipid molecule (*z*), within a series of distance windows. Reliable results depend on effective sampling of conformational space for this special lipid molecule which presents a challenge for molecules as large and flexible as the ILs depicted in Figure 1. In the following we will refer to calculations for each type of system by the label used for the IL in Figure 1, i.e., ALC-0315 for COMIRNATY*, MC3 (from DLin-MC3-DMA) for Onpattro*, and SM-102 for Spikevax* where * indicates that the PEG lipids and RNA were left out as well as rounding to an integer number of molecules. Lipid A refers to the variant of the COMIRNATY* formulation with that lipid instead of ALC-0315.

Initial equilibration of each bilayer utilized the standard relaxation protocol used in Schrödinger Suite’s implementation of Desmond^30,31^ within Maestro.^32^ This protocol consisted of a Brownian dynamics step, a canonical ensemble step, and three subsequent isobaric–isothermal ensemble simulations. This process and all other simulations in the current work employed the OPLS4 force field^33^ with the SPC water model.^34^ A 1 μs NPγT molecular dynamics (MD) simulation at 310.15 K, 1.013 bar, and 0 surface tension was then performed to produce a well-relaxed bilayer.

A lipid buried within the membrane is selected for umbrella sampling (see the SI for more information). For all of the umbrella sampling calculations the membrane is restrained to have a net z position of 0 (see the SI for additional information). Starting configurations for the umbrella sampling windows were created for this lipid starting from the two windows centered above and below the initial lipid position (as defined by the headgroup N atom). The system was relaxed in each window for 10 ns before initiating the relaxation in the following window further out. Adjacent windows were separated by 1.0 Å, and each relaxation was carried out with an applied harmonic potential of 2.0 kcal/mol/Å^2^ in the *z* direction and 1.0 kcal/mol/ Å^2^ in the *x* and *y* directions. Each relaxed window was sampled for 100 ns with the same harmonic potentials applied to the N atom in the IL as was used during relaxation. The relaxations and the sampling runs utilized NPγT MD under the same conditions used for the 1 μs simulation. The range of *z* values sampled was −2 to 50 Å for all systems except MC3 where the range was −2 to 55 Å, covering the range of *z* values from the central region of the membrane to bulk aqueous solution. The distribution of z values visited was then calculated for each window for times between 30 and 100 ns and, utilizing the weighed histogram analysis method (WHAM),^35^ the unbiased potential of mean force (PMF) was determined at a 0.2 Å resolution for each system in Figure 1. To obtain more accurate estimates and statistics, we have sampled each system in Figure 1 across six replicas for each of the positive and neutral forms of the ILs, by selecting different lipids within the equilibrated bilayer for the umbrella sampling. For each system the PMF was smoothed over 11 adjacent values and then shifted to 0 energy at large bilayer–lipid separations.

Δp*K*_a_ is calculated using
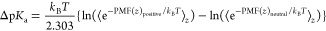
1where the averages run over *z* ranges from 0 (the center of membrane) to where the PMF no longer deviates from the bulk value (for ALC-0315, 45.5 Å; for Lipid A, 49 Å; for MC3, 52 Å; for SM-102, 40 Å), *k*_B_ is the Boltzmann constant, and *T* is the temperature in kelvin. p*K*_a_^A^ is given by
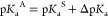
All post-processing of the PMF curves was carried out using Google sheets.

Despite the simplicity of this methodology, the overall accuracy also relies on having a highly accurate p*K*_a_^S^ value for each of the lipids. Unfortunately, due to both common lipid solubility challenges and the limited accuracy of p*K*_a_^S^ prediction methods, care needs to be taken in selecting p*K*_a_^S^ values. We used the recently created ML-based version of Epik^36^ which has been extensively parametrized for a wide range of organic molecules in water to calculate p*K*_a_^S^.

## Results and Discussion

**Figure 2:**
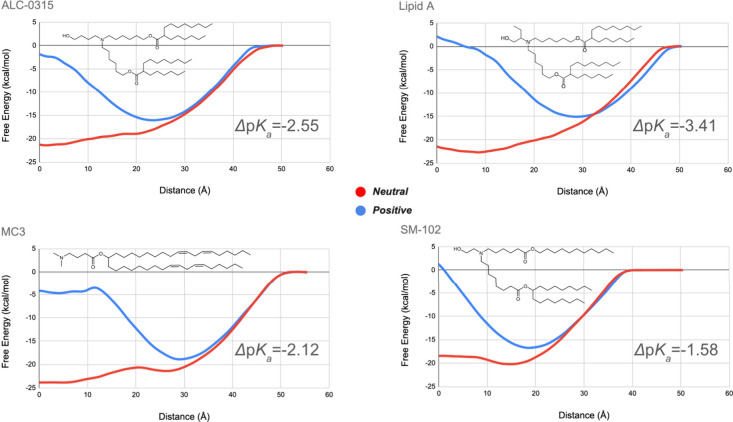
PMF for charged (blue) and neutral (red) lipids, averaged over all 6 replicates relative to the center of the bilayer (distance = 0) out into bulk aqueous solution, where these values become flat. The Δp*K*_a_ calculated for each of the lipids is included for each formulation. The PMF plots for the replicates are provided in the Supporting Information.

As shown in Figure 2, the PMF for the neutral and charged lipids are quite different. The MC3 system required greater distances from the center of the bilayer in order to obtain flat free energy curves than the other 3 systems. All in their neutral form have broad, deep minima in the bilayer with MC3 having the strongest favorable free energy for integration of the lipid molecules. The positive forms have deep minima within the headgroup regions in the bilayer for each formulation at distances ranging from 18 to 28 Å. At smaller distances the free energy rises approaching or exceeding that for bulk water (0 kcal/mol) in the center of the bilayer, reflecting the free energy cost for burying the charged headgroup inside a low-dielectric region. The minimum for the positive form is deepest for MC3. These minima are weaker and narrower than the corresponding wide and flat low free energy regions for the neutral lipids in all cases. The stronger overall binding of the neutral forms leads to the effective drop in the p*K*_a_^A^ relative to the p*K*_a_^S^ value (i.e., a negative Δp*K*_a_ value). For MC3, the effect of the strong minima largely cancels out yielding one of the smaller |Δp*K*_a_|. Published MD simulations of bilayers containing ILs indicate that the neutral head groups exist and perhaps favor burial inside membranes while the positive head groups remain on the surface of lipid structures and retain their exposure to water.^37−39^ The contrast between the positioning of the neutral and charged ILs seems most extreme for MC3 in our studies.

Table 1 contains the calculated p*K*_a_^S^, Δp*K*_a_, and p*K*_a_^A^ values for all 4 lipid systems along with experimental p*K*_a_^A^ values. The literature lists similar experimental values for ALC-0315,^10,13,17^ MC3^10,11,13,17^ and SM-102.^10,13,17,18^ Interestingly for the three lipids used in therapeutics, the trend in the p*K*_a_^A^ values for both our calculations and the experimental values orders ALC-0315 < MC3 < SM-102 which is the opposite of the trend for the p*K*_a_^S^ values, i.e., the shift in the p*K*_*a*_ values is anticorrelated with the p*K*_a_^S^ values for these formulations. Lipid A does not fit this trend having a midrange p*K*_a_^S^ value and the most negative Δp*K*_a_ value yielding the lowest p*K*_a_^A^ value.

**Table 1:** p*K*_a_^S^, Δp*K*_a_, and p*K*_a_^A^ Values^a^

			p*K*_a_^A^	
Lipid/formulation	p*K*_a_^S^	Δp*K*_a_	Calcd	Expt
ALC-0315	9.26	–2.55	6.71	6.11
Lipid A	9.01	–3.41	5.60	4.67
MC3	9.00	–2.12	6.88	6.44
SM-102	8.56	–1.58	6.98	6.53

a Experiment refers to experimental p*K*_a_^*A*^ values measured for LNPs from Ref (41) and a description of the assay is available in the SI.

As a check we also equilibrated the Lipid-A system for 2 μs prior to repeating all of the umbrella calculations for the replicates for the positive and neutral ILs. The calculated Δp*K*_a_ value was −3.38 as compared to the value of −3.41 reported in Table 1. Since the difference between these values is much less than the standard deviation (0.42 p*K*_a_ units) the 1 μs equilibration time seems adequate. Similarly we recalculated the Δp*K*_*a*_ for ALC-0315 using the final structures from the initial umbrella sampling windows as input structures for a subsequent umbrella sampling calculation (i.e., each of these windows were effectively equilibrated for an additional 100 ns). The calculated Δp*K*_*a*_ value was −2.71 as compared to the value of −2.55 reported in Table 1. Since the difference between these values is less than the standard deviation (0.44 p*K*_*a*_ units) then the sampling of the lipid conformations in the 100 ns sampling runs seems adequate. As an additional check, conformations of ALC-0315 for the first replicate at the start and end of sampling runs were compared and found to be quite different (see SI Figure S4) which is consistent with significant lateral diffusion within the membrane as demonstrated in the SI. Figure S5 provides the distributions of distances sampled for one of the replicates and demonstrates good overlap of these distributions for adjacent umbrella windows. The SI also has a comparison for the surface area per lipid calculated from information on a MC3 formulation derived from experiment^40^ with the surface area using the equilibrated membrane in the current work.

**Figure 3:**
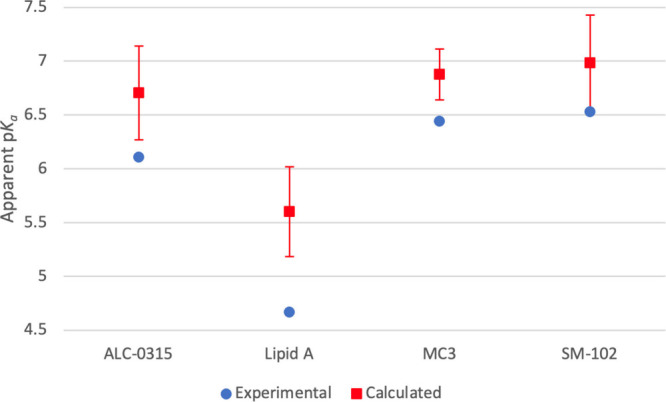
Calculated and experimental apparent p*K*_a_ value trends. The experimental values are shown in blue, while the calculated values are red. The error bars for the calculated values represent one standard deviation of the average.

Figure 3 is a plot of the experimental and calculated p*K*_*a*_^*A*^ values. Error bars are the standard deviation of the average value calculated from the spread in calculated Δp*K*_*a*_ values for the six replicates. The size of the error bars could be reduced by adding more replicas or running longer umbrella sampling simulations. Overall, the trend in calculated as compared to experimental p*K*_*a*_^*A*^ values is well reproduced with a *R*^2^ value of 0.998 (for the 3 commercialized formulations *R*^*2*^ has a value of 0.971). The calculated values are somewhat higher and span a smaller range.

## Conclusion

In summary, we have employed a methodology for predicting the p*K*_a_ shift for a lipid between bulk aqueous solution and a lipid bilayer as a stand-in for the environment within an LNP. This shift can be combined with the bulk aqueous solution p*K*_a_ value to yield apparent p*K*_a_ values for the IL for LNP formulations that look promising for classifying IL candidates as potentially promising or unlikely to have useful p*K*_a_^A^ values. Since we omit PEG lipids and mRNA from these calculations the resulting values are likely most relevant for ILs not associated with mRNA in situations where the PEG lipids have detached from the LNP. To our knowledge, this work represents one of the first reported nonexperimental methodology for calculating the apparent p*K*_a_ values of ILs in LNPs. There are other ways that these values could potentially be calculated including absolute binding free energies using FEP,^42^ metadynamics,^43^ or constant pH simulations using molecular dynamics^44−46^ or Monte Carlo techniques.^20^ However, to the best of our knowledge, these have not been explored yet for this specific application with the exception of Monte Carlo techniques.^20^

We plan to continue to expand and verify this new method with more subtle variations in lipid structure and also examine some of the underlying, collective structural features that influence the p*K*_*a*_ values in a future work. This methodology can be applied directly to new formulations and is expected to expedite the development of new nanoparticle systems for therapeutic delivery.

## Supporting Information Available

System composition for each formulation; PMF plots and calculated p*K*_a_^A^ values; bilayer construction method; umbrella sampling calculation method description; conformational changes visual characterizations; umbrella sampling run histograms; description of the TNS fluorescence assay (PDF)
Scripts and input structure files for building and running these jobs in conjunction with Schrödinger Release 23.4 (ZIP)
