# Correction to “Calculating Apparent p*K*_a_ Values of Ionizable Lipids in Lipid Nanoparticles”

**DOI:** 10.1021/acs.molpharmaceut.5c00402

**Published:** 2025-04-08

**Authors:** Nicholas B. Hamilton, Steve Arns, Mee Shelley, Irene Bechis, John C. Shelley

**Affiliations:** † Schrödinger, Inc., 101 SW Main St., Suite 1300, Portland Oregon 97204, United States; ‡ Acuitas Therapeutics, 6190 Agronomy Road, Suite 405, Vancouver, BC Canada, V6T 1Z3; § Schrödinger, GmbH, Glücksteinallee 25, 68163 Mannheim, Germany

This supplement is to correct a mistake in an equation and corresponding numeric values within the original manuscript.

In the original manuscript the equation used for calculating the shift in the apparent p*K*_a_ (p*K*_a_^A^) values from the p*K*_a_ value in bulk water (p*K*_a_^S^), Δp*K*_a_, incorrectly included a term, *k*_B_*T*, rather than just 1. The correct form for eq 1 is
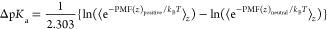
1The corrected Δp*K*_a_ and apparent p*K*_a_^A^ values are given in Table 1 and plotted in a revised Figure 3 below.

**Table 1:** p*K*_a_^S^, Δp*K*_a_ and p*K*_a_^A^ Values^a^

			p*K*_*a*_^*A*^	
Lipid/formulation	p*K*_*a*_^*S*^	Δp*K*_*a*_	Calcd	Expt
ALC-0315	9.26	–4.15	5.11	6.11
Lipid A	9.01	–5.53	3.48	4.67
MC3	9.00	–3.44	5.56	6.44
SM-102	8.56	–2.56	6.00	6.53

a Experiment refers to experimental p*K*_a_^A^ values measured for LNPs from ref (1) and a description of the assay is available in the Supporting Information.

**Figure 3:**
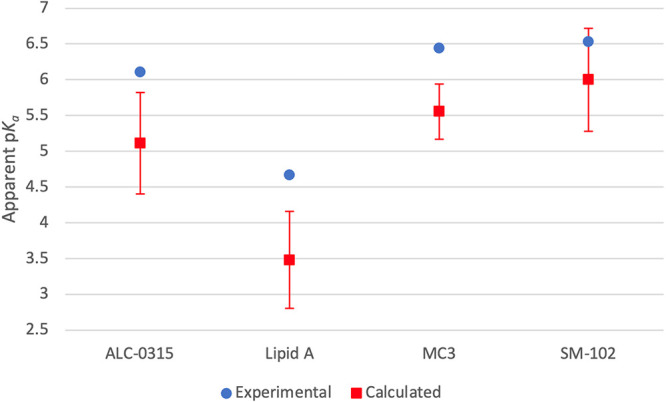
Calculated and experimental apparent p*K*_a_ value trends. The experimental values are shown in blue, while the calculated values are red. The error bars for the calculated values represent one standard deviation of the average.

The calculated p*K*_a_^A^ values are now systematically lower than the experimental values. Overall, the trend in calculated as compared to experimental p*K*_a_^A^ values is well reproduced with a *R*^2^ value of 0.997 (for the 3 commercialized formulations *R*^2^ has a value of 0.996). Despite the changes in the numeric results, the conclusions for the article remain unchanged. We apologize to the readers for the mistake in eq 1 and are grateful to the journal for allowing us to publish this correction.

## References

[ref1] The p*K*_*a*_ value for Lipid A was measured by Acuitas using a TNS fluorescence assay. Please see the Supporting Information of the original manuscript for additional information.

