# Supplementary material for: Calculating Apparent pKa Values of Ionizable Lipids in Lipid Nanoparticles
Source: Mol Pharm. 2024 Dec 10;22(1):588–93. doi: 10.1021/acs.molpharmaceut.4c00426 (PMC11707724; doi:10.1021/acs.molpharmaceut.4c00426)
Supplement: Supplementary file 1 — mp4c00426_si_001.pdf [file mp4c00426_si_001.pdf]

Supporting Information:

Calculating Apparent pK_a Values of Ionizable Lipids in Lipid Nanoparticles

Nicholas B. Hamilton[1], Steve Arns[2], Mee Shelley[1], Irene Bechis[3], and John C. Shelley[1*]

[1]*Schrödinger, Inc., 101 SW Main St., Suite 1300, Portland OR 97204, USA*

[2]*Acuitas Therapeutics, 6190 Agronomy Road, Suite 405, Vancouver, BC, Canada, V6T 1Z3*

[3]*Schrödinger, GmbH, Glücksteinallee 25, 68163 Mannheim, Germany*

List of Contents

Composition of Systems Simulated

Plots of the Potential of Mean Force for All Calculations

Method for constructing bilayers

Description of Umbrella sampling techniques used

- Selecting and centering lipids prior to Umbrella sampling
- Additional information on umbrella sampling

Conformation changes during the sampling run

Histograms from an umbrella sampling calculation

Description of the TNS fluorescence assay for measuring p$K_a{}^A$

Accompanying this SI text file is a tgz file containing scripts and input structure files for use with Schrödinger Release 23-4. See the How_to_run_LNP_pKa text file within that tgz file for additional information.

Composition of Systems Simulated

Table S1. Number of molecules of each type for the formulations simulated

Component	ALC-0315	Lipid A	MC3	SM-102
ionizable lipid	48	48	48	48
DSPC	10	10	10	10
Cholesterol	42	44	40	40
Na^+ ions	40	40	40	40
Cl^- ions	64	64	64	64
water	12200	12200	12200	10674

Plots of the Potential of Mean Force for All Calculations

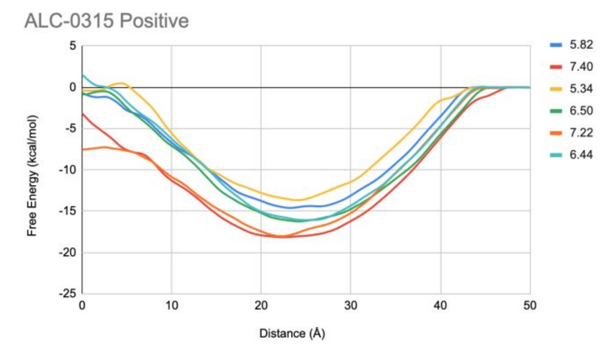

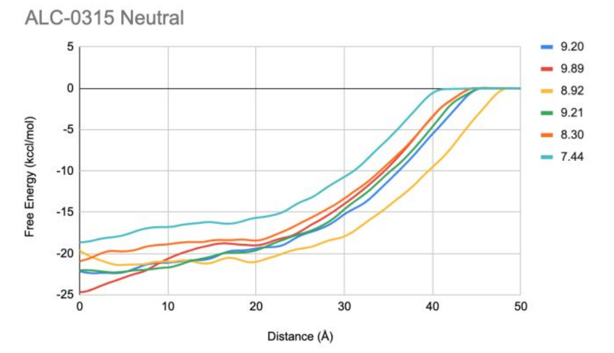

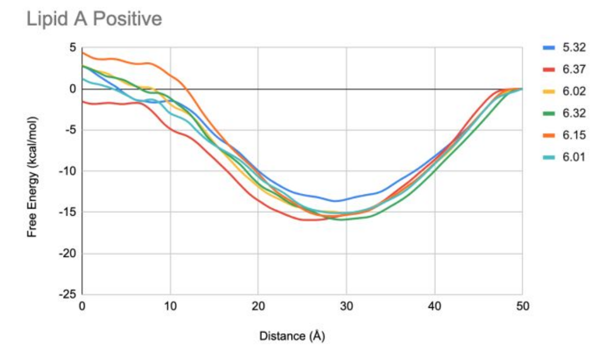

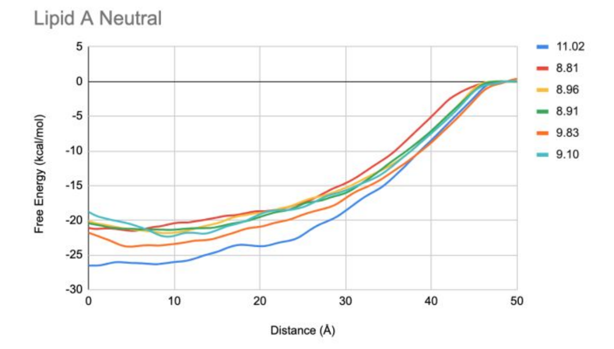

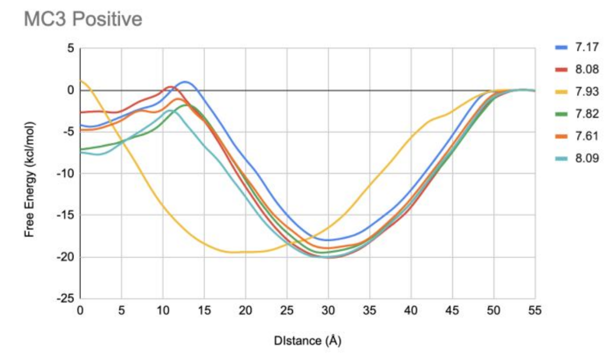

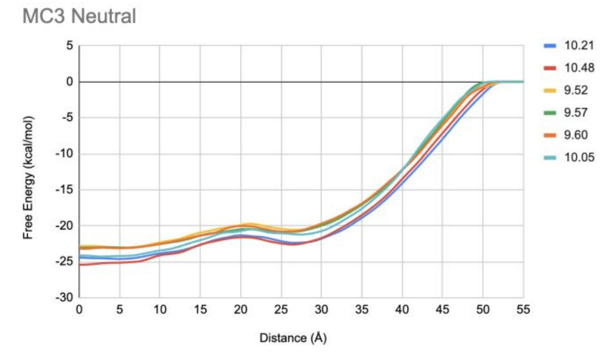

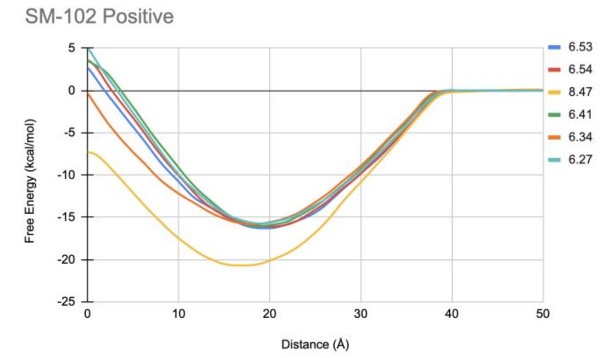

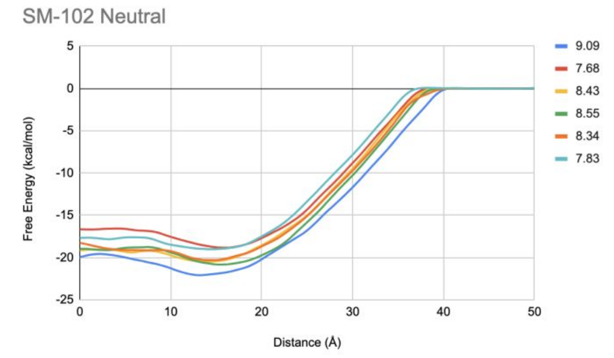

Figure S1. Potentials of mean force from each replicate calculation for positively charged and neutral ligands. The labels are the terms $\frac{k_B T}{2.303} ln\left(< e^{-\frac{PMF(z)_{positive}}{k_B T}} >_z\right)$ and $\frac{k_B T}{2.303} ln\left(< e^{-\frac{PMF(z)_{neutral}}{k_B T}} >_z\right)$ for each plot which have the same units as pK_a.

Method for constructing bilayers

Packmol is used to construct the initial bilayer structure

Packmol was used to construct the bilayers including the aqueous solution with a salt concentration of 0.15 M NaCl. The Packmol input script used for creating each system was itself created using a python script liquid_builder.py using instructions in a system-dependent json file (build.json).

For each lipid the build.json file gives its name (which is also used as basis for the name of a Maestro structure file containing the 3D structure for a lipid), one or more atoms considered as hydrophilic ("atom1") and one or more atoms considered as hydrophobic ("atom2") as well as the mol % of the lipid in the membrane. The neutral and positive forms of the ionizable lipid are listed separately in build.json. The last lipid listed should be the positive form as this is used to determine the number of Cl- counter-ions to include. The builder.py script is run with build.json as the only argument and it produces an 'inp' input file for the wrapper for Packmol and a sh script to run the Packmol job.

In the build process Packmol is instructed to build a system within an orthorhombic region 85 Å X 85 Å X 150 Å with the z direction perpendicular to the bilayer. In the rest of this description the middle of the bilayer is assumed to be at z=0. The system is constructed compositionally symmetric w.r.t. z=0 although the coordinates for the molecules in the 2 halves of the system are different. The system is constructed with:

- Two layers of water each containing 6100 water molecules are placed between 34.11 and 75 Å away from the middle of the bilayer
- 20 Na+ in each of the same regions as water was placed
- 20 + ½ (the number of positively charged ILs) Cl- ion in each of the same regions as water was placed
- ½ the number of each type of lipid is placed on each side of the bilayer with all hydrophilic atoms further away from the middle of the bilayer than 24.95 Å and all hydrophobic atoms closer to the middle of the bilayer than 31.11 Å.

Checking for ring spears

While Packmol can be very efficient at creating bilayer systems sometimes the systems constructed with Packmol have molecules with significant van der Walls overlap. This is problematic when parts of one molecule pass through rings in another molecule, in this case cholesterol. To reduce the occurrence of this problem the initial bilayer system was constructed with a low density. However, ring spears could still occur so the systems were checked with the Locate Rings and Spears tool in the Materials Maestro GUI. If ring spears

were identified in a particular system some atoms were interactively dragged to new locations and locally energy minimized using the build tool in Maestro.

Scaling the x and y coordinates of the system
The system was scaled in the x y coordinates using the script: scale_xy_z.py by a factor of 0.647 to obtain a system with a surface area per lipid molecule (assuming 100 lipids total) of 60.5 Å^2.

Selecting and centering lipids prior to Umbrella sampling
ILs were selected from the final structure from the 1 μs relaxation simulation manually using the trajectory viewer in Materials Maestro with a bias towards selecting lipids with diverse positions of the IL head-group N atom relative to the center of the bilayer.

The umbrella sampling script assumes that the bilayers is close to being centered around z = 0 and that the N atom in the head-group of the IL is centered around x = y = 0. This was accomplished interactively within Materials Maestro when viewing the last frame in the trajectory by:

1. Centering the DSPC molecules
2. Identifying an atom in the system (refer to as counter N atom) with coordinates approximately (x, y, -z) where (x, y, z) are the coordinates for the N atom
3. Recentering the system on the N atom and the counter N atom.
4. If the z coordinate for the N atom is less than 0 rotate the system about the y axis by 180° (using the script rotate_y_180.py), so that the z coordinate used in the subsequent umbrella sampling calculations is positive.

Additional information on umbrella sampling

Restraining the membrane
The membrane was restrained using four atoms (atom numbers within the molecule: 1, 10, 45, and 54) in the DSPC molecules as shown in Figure S2 below.
During the relaxation process each of the atoms in each of the DSPC molecules is restrained relative to its initial position using a harmonic potential with a weak force constant of 0.05 kcal/mol/Å^2. During sampling the center of mass of all of these atoms across all DSPC molecules is restrained in z relative to z=0 with a harmonic potential with a force constant of 5 kcal/mol/Å^2.

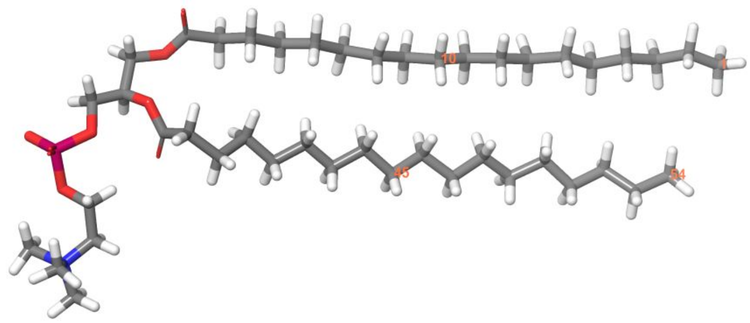

Figure S 2. The atoms within the DSPC molecule that are restrained during the umbrella sampling calculations relaxation and are used to calculate the center of mass z position for positioning the membrane during umbrella sampling calculations are labeled by atom number within the molecule.

Overview of the Umbrella sampling process

The overall umbrella sampling process is described in the article and depicted in Fig. S3. At the end of the 1 μs simulation to equilibrate the membrane, lipid molecules are chosen for umbrella sampling calculations. A separate umbrella sampling job is run for each lipid selected. The lipid is centered in the upper leaflet as described above as shown in Fig. S3a. The version of the system with the structure centered is processed using the Prepare for MD tool in Maestro to produce a cms input structure file for Desmond. Information about this lipid is stored in a .json file which controls how the umbrella sampling calculation is done.

These lipid-focused umbrella sampling involves determining distance distributions for the N atom in the lipid head-group in separate simulations each sampling a different range of distances as limited by an applied harmonic potential. The minimum for the harmonic potential for each window ranges from a specified minimum z value to a specified maximum z value at 1 Å intervals. The overall calculation proceeds in two broad stages:

1. Generating starting structures for each umbrella sampling window
2. Running the umbrella sampling calculation itself

The starting structures are generated as described in the article and depicted in Fig. S3b. Briefly, given a centered lipid structure the two windows with minima in the harmonic restraint potentials immediately above and below the initial z value for the N atom in the lipid head-group are identified and the relaxation jobs which consists of a 10 ns Desmond simulations for those windows with the restraint potentials are launched (e.g., if the initial position was 24.1 Å these are the windows with harmonic potential minima at 24 and 25 Å). When either of these jobs finishes the relaxation job for the next window further out from the initial z position is launched using the final structure from the just completed relaxation. This process continues in each direction until relaxed structures.

The umbrella sampling calculation is run as shown in Fig. S3c. This involves a separate 100 ns Desmond simulation for each window starting from the relaxed structure for that window. When each such job finishes the distribution of distances sampled for times between 30 and 100 ns is determined. These distributions are then collectively subjected to WHAM analysis to determine the PMF. The WHAM process is iterative and proceeds until the largest change in

adjacent tabulated PMF points between consecutive iterations is less than a threshold of 1.0^{-6} kcal/mol or until 4,000 iterations have been completed.

The overall umbrella sampling process encompassing the equilibration of the windows, sampling of the windows, histogram generation and WHAM analysis can be carried out in a single run using the umbrella_sampling_1D.py. In the current case we separated out the relaxation step from the rest of the umbrella sampling calculations because that worked better on the queuing systems available to us.

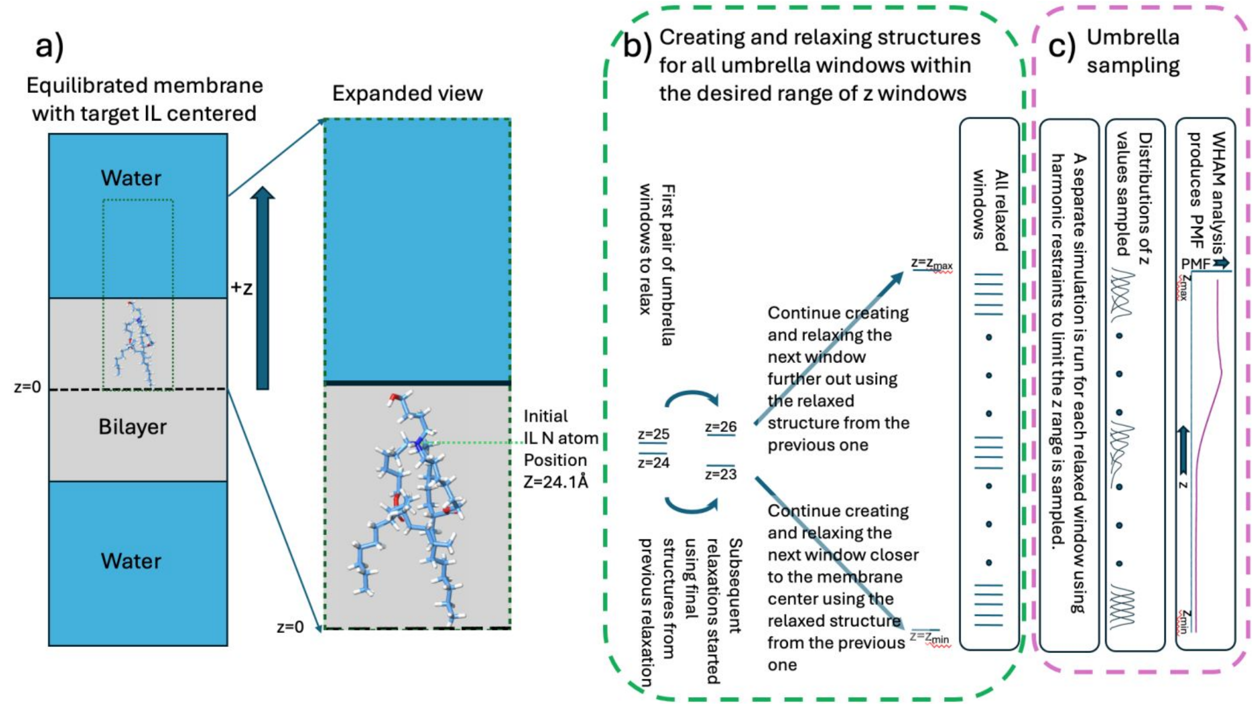

Figure S 3. The umbrella sampling process

<u>Conformation changes during the sampling run</u>

Figure S 4 shows the conformations for the first replicate of ALC-0315 for (a) the neutral IL that was subject to umbrella sampling and (b) the positive IL that was subject to umbrella sampling at the start and end of the sampling run. The windows with a minimum in the applied harmonic potential at 4.5 Å for the neutral and 24.5 Å for the positive IL were chosen because they roughly coincide with the minimums in the PMFs for these two species as show in Fig. 2. One can see the large-scale differences in conformation for each of these cases.

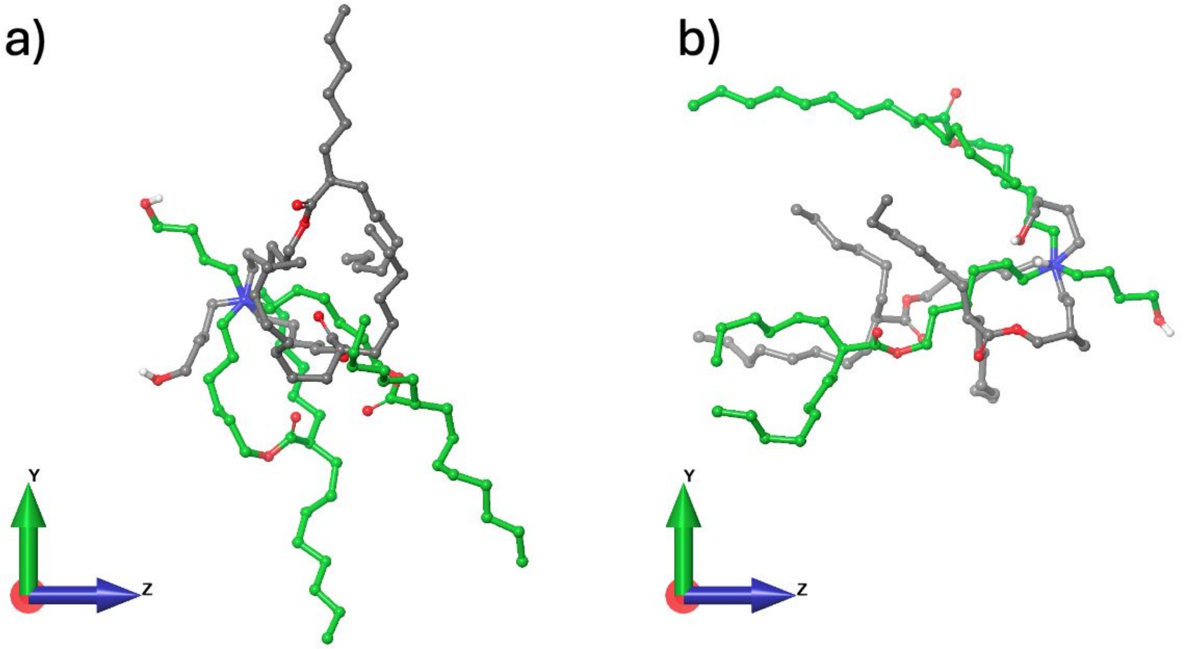

Figure S 4 Conformations of ALC-0315 used in the umbrella sampling runs for the first replicate at the beginning (grey carbons) and end (green carbons) of the sampling runs a) for the neutral IL for the umbrella window with the minimum of the harmonic potential at 4.5 Å and b) for the positively charged IL for the umbrella window with the minimum of the harmonic potential at 24.5 Å. The head-group N atoms were translated to coincide and the non-polar hydrogen atoms have been hidden to facilitate comparing the changes in conformation of these ILs.

Histograms from an umbrella sampling calculation

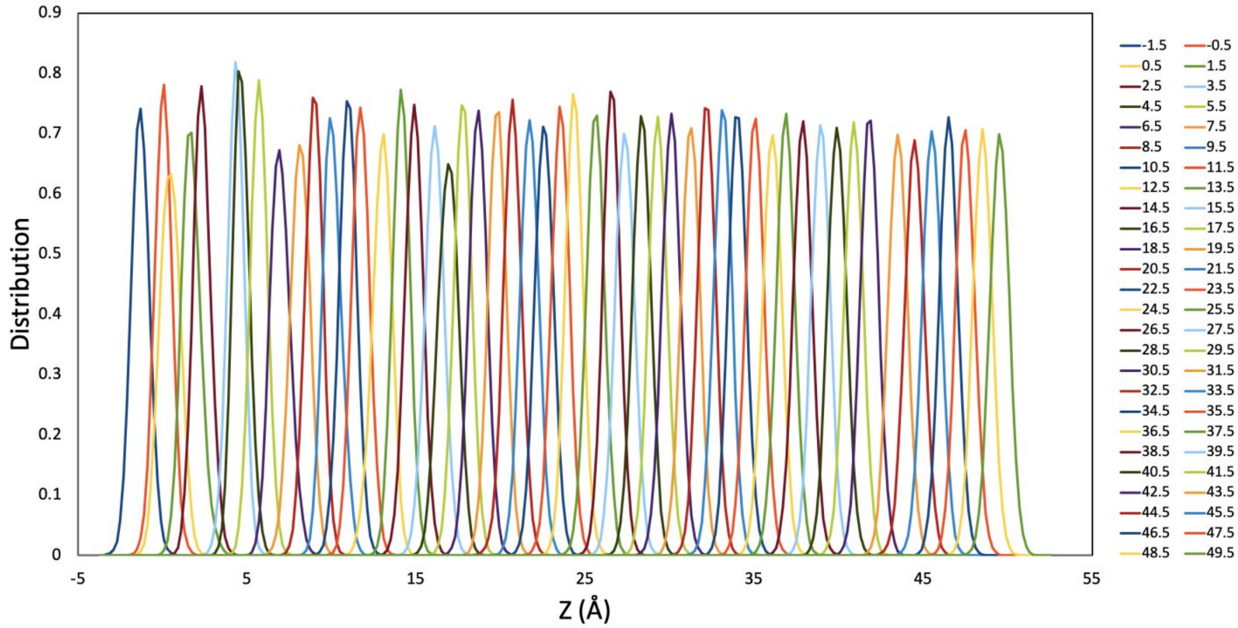

Figure S 5 Histograms of distributions sampled during umbrella sampling run for the positive ALC-0315 for the first replicate. Each curve is from a different umbrella sampling subjob with an applied harmonic potential at the distance indicated in the legend.

Diffusion within the membrane

The Materials Science Diffusion Coefficient calculation tool was used to calculate the lateral (i.e., within the plane of the membrane) diffusion constant of the center of mass of the positively charged ALC-0315 lipid using the starting structure from an umbrella sampling calculation (i.e., after the 1 μs relaxation simulation) based upon the mean squared displayment.[1] The calculation was run for the same length as an umbrella sampling window: 100 ns and is based upon the diffusion of all lipids of this type. The value obtained depended somewhat on the time frame for the linear fit to the mean squared displacement, namely: 4.25 X 10^{-10} m^2/s for 0.01-10 ns, 4.68 X 10^{-10} m^2/s for 0.01-20 ns, 4.14 X 10^{-10} m^2/s for 5-10 ns, and 4.94 X 10^{-10} m^2/s for 5 to 20 ns. These values correspond to a typical, positively charged, ALC-0315 lipid undergoing significant motion (> 60 Å) laterally within the membrane over the course of a 100 ns simulation. This scale of motion is consistent with the large shifts in conformation depicted in Fig. S 4 plus the width (~ 2 Å) and smoothness of the distributions (well sampled) from the individual umbrella sampling windows in Fig. S 5.

Description of the TNS fluorescence assay for measuring p$K_a{}^A$

The p$K_a{}^A$ of each lipid is determined in LNPs using an assay based on fluorescence of 2-(p-toluidino)-6-napthalene sulfonic acid (TNS). Lipid nanoparticles were prepared in PBS at a concentration of 0.4 mM total lipid using an in-line process. TNS is prepared as a 100 μM stock solution in distilled water. Vesicles are diluted to 24 μM lipid in 2 mL of buffered solutions containing 10 mM HEPES, 10 mM MES, 10 mM ammonium acetate, and 130 mM NaCl, where the pH ranged from 2.5 to 11. An aliquot of the TNS solution is added to give a final concentration of 1 μM and following vortex mixing fluorescence intensity is measured at room temperature in a SLM Aminco Series 2 Luminescence Spectrophotometer using excitation and emission wavelengths of 321 nm and 445 nm. A sigmoidal best fit analysis is applied to the fluorescence data and the p$K_a{}^A$ is measured as the pH giving rise to half-maximal fluorescence intensity.

Comparision of surface areas per lipid

There may be some interest in comparing the surface area from our calculations with those inferred from experiment, in particular, those from Ref. 2 in which SAXS, SANS and DSL were used to characterize the structure of an MC3 based-LNP with an overall composition (DSPC/cholesterol/MC3/DMPE-PEG = 10/38.5/50/1.5 mol %) similar to that used in our study (DSPC/cholesterol/MC3 = 10.2/40.8/49 mol %). We note that the authors of Ref. 2 did not attempt to calculate a surface area per lipid so in this section we will try to estimate this area using additional calculations based upon their data. There are a number of reasons why our results may not match well with those derived from these experiments including:

(1) In Ref. 2 the analysis reasonably breaks the LNP structure down a core and a shell and it is not clear how to determine the surface area for the structures that form in the core. As a result this comparison will be focused on the shell which seems to have a fairly different composition than the LNP as a whole (see below).
(2) The analysis of the experimental data employs a spherical model which averages over any inherent non-sphericity of the LNP and fluctuations in the surface of the LNP. Such averaging will lead to an underestimation of the surface area.

(3) The experimental analysis is based on an assumption that 100 % of the DSPC is in the shell. While localization of much of the DSPC to the shell seems reasonable in this formulation it seems plausible that some of it is also in the core and that may in turn affect the surface area estimates.
(4) Ref. 2 used a pH of 7.4 which means that most of the MC3 will not be protonated (experimental $\mathrm{p}K_a{}^A$ = 6.44) while our calculations include equal amounts of protonated and non-protonated MC3 lipids.
(5) The nature of the outer shell of the LNP is somewhat ill-understood in general. There seems to be variety of characterizations in the literature regarding the shell as a monolayer or a bilayer or perhaps something more complex. The geometry matters when calculating the surface area. The shell thickness reported in Table 2 of Ref. 2, 6 $\pm$ 1 nm (60 Å $\pm$ 10 Å), is roughly consistent with the thickness of a bilayer in our simulations.
(6) The ~17% uncertainty in the experimental shell thickness and ~7 % uncertainty in the LNP radius reported in Table 2 of Ref. 2. means that we should not expect close agreement between the surface area from our simulations and those from experiment.

Ref. 2 reports the volume fraction of components in the shell as: DSPC/cholesterol/MC3/DMPE-PEG = 26:33:38:3. We calculate the volumes of these lipid molecules as: DSPC/cholesterol/MC3/DMPE-PEG = 1370/ 721/1291/1022 $Å^3$/molecule using the Coarse-grained Force Field Builder in the Materials Science suite[3]. For the DMPE-PEG we leave out the volume of the PEG groups since as depicted in Fig. 1 E in Ref. 2, they extend into solution. This lipid is a minor component in any case and thus this assumption should not strongly affect this calculation. Dividing the volume fractions by the corresponding molecular volumes and normalizing gives a shell composition of: DSPC/chol/MC3/DMPE-PEG = 19.5/ 47.1/30.3/3.0 mol % which is quite different from that used in our calculations: (DSPC/cholesterol/MC3 = 10.2/40.8/49 mol %).

The surface area per lipid can be estimated using two near-extreme models for the shell geometry:
(1) It is a monolayer with all solvent-exposed polar groups facing the outer surface
(2) It is a bilayer with an inner and outer surface
and we expect that the actual surface area would likely be between these two values.

The outer surface area can be estimated using the average LNP radius from Table 2 of Ref. 2 (330 Å) as: 1,368,477 $Å^2$.
The radial location of the inner surface of a bilayer-like shell would be located at the LNP radius – the shell thickness (330 Å $-$ 60 Å $=$ 270 Å) as: 916,088 $Å^2$.

The number of lipids present in the shell can be estimated using the volume fractions of the lipids in the shell and the shell volume from Ref. 2 along with our estimated lipid volumes per molecule as: DSPC/cholesterol/MC3/DMPE-PEG = 12,805/30,881/19,859/1,981 for a total of: 65,526.

Therefore the surface area for a monolayer model of the shell would be: 21 $Å^2$ while the surface area for a bilayer model of the shell would be: 35 $Å^2$

We carried out a 100 ns simulation of the 1 μs MC3 formulation bilayer used in our calculations in the same way as used in the umbrella sampling runs except that no restraints were applied. The average surface area for one side of the bilayer was calculated as: 1660.25 Å^2 (the standard deviation of the average surface area was 0.18 Å^2). The simulation has two surfaces and 98 lipids total giving an average surface area per lipid as: 33.9 Å^2. This is intermediate between the monolayer (21 Å^2) and bilayer estimates (35 Å^2) for the surface area per lipid made above based upon the data from Ref. 2 and somewhat closer to the bilayer value.

References

(1) B. Smit and D. Frenkel, Understanding Molecular Simulation, Second Edition (Academic Press, 2002).
(2) F. Sebastiani, M. Y. Arteta, M. Lerche, L. Porcar, *et al.* Apolipoprotein E Binding Drives Structural and Compositional Rearrangement of mRNA-Containing Lipid Nanoparticles. *ACS Nano* **2021** *15* (4), 6709-6722. DOI: 10.1021/acsnano.0c10064
(3) Schrödinger Release 2023-2: Materials Science Suite; 2023.
