# Supplementary material for: Calculating Apparent pKa Values of Ionizable Lipids in Lipid Nanoparticles
Source: Mol Pharm. 2024 Dec 10;22(1):588–93. doi: 10.1021/acs.molpharmaceut.4c00426 (PMC11707724; doi:10.1021/acs.molpharmaceut.4c00426)
Supplement: Supplementary file 2 — mp4c00426_si_002.zip [file mp4c00426_si_002.zip › SI_input_files_and_scripts/How_to_run_LNP_pKa.pdf]

**Preparation:**

- Open "lnp-run.tgz" to find 5 directories: "input-files" containing input files, and "bilayer_builder", "preparing_structure", "run_1us", "umbrella_sampling" corresponding to the 4 steps described below. Copy a pair of lipid files for each system (for example, ALC-0315-N.mae and ALC-0315-P.mae) to the "bilayer_builder" directory and follow the steps described below. Lipid files are referred to as ILN (ionizable lipid, neutral) and ILP (ionizable lipid, protonated) below.
- Place "scripts.tgz" on a machine that will be used for MD simulations. Open "scripts.tgz" and note the path to the "scripts" directory.
- These instructions are for the 23-4 or later releases of the Schrödinger software.

**Outline:**

1. Build a LNP bilayer
2. Prepare a structure for MD
3. Run 1μs MD simulation
4. Umbrella sampling

**Step 1: Build a LNP bilayer** (directory: bilayer_builder)

- Goal: Build a bilayer based on an input .json file and structure files for the components
- Tasks:
    - Copy a pair of lipid files from the "input-files" directory. For example, they are ALC-0315-N.mae (ILN) and ALC-0315-P.mae (ILP).
    - Edit “build.json” to specify
        - names of 2 lipid files
        - atom1 (polar end) and atom2 (hydrophobic ends): atom numbers (residue names = ILN and ILP in mae files)
        - < jobname>
        - Note: You can copy a build.json file provided for each of the 4 lipid systems from the "input-files" directory (e.g. copy "build.json_for_ALC-0315" to build.json to work on the ALC-0315 system). Please note the jobname specified in the .json file.
    - $SCHRODINGER/run liquid_builder.py
        - <jobname>.inp and <jobname>.sh will be created
    - Run <jobname>.sh
        - <jobname>_out.mae (containing the bilayer) will be created
        - Import <jobname>_out.mae into Maestro and proceed to the next step (prepare a structure for MD)
- Notes
    - build.json specifies lipid filenames as well as the desired mol%s for the system
    - Component .mae files (CHOL, DSPC, NA, CL-, H2O, and lipids) should be present in the directory
    - Atom numbers are used to determine the orientation of lipid molecules
    - The mol%s are converted to the appropriate number of molecules

- The system will be built at 85 A x 85 A to reduce ring spears and problems with overlapping molecules
- liquid_builder.py will use build.json to create the input file and command to run the Structured Liquid builder based on the 'jobname' in build.json

**Step 2: Prepare a structure for MD** (directory: preparing_structure)

- Goal: Fix ring spears (if any) and assign force field (OPLS4)
- Tasks:
  - Change Maestro Working Directory to “preparing_structure”
  - Open the “Locate Ring and Spears” panel and click “Locate” (with <jobname>_out.mae from Step 1 in the workspace)
  - Note: If the built lipid bilayer system has >25000 atoms, locating ring spears may take time, press “continue” in the popup GUI and wait until locating ring spears is done.
  - If ring spears are found, resolve manually (See below for the recommended procedure). It will be rare to find ring spears due to recent improvements to the protocol, but it is very important to check. Simulation results will not be useful if any ring spear is present.
  - Open “Prepare for Molecular Dynamics”
  - Toggle ON “Redistribute heavy atom mass to hydrogens” and click “Run"
  - Scale the system using the script scale_xy_z.py
    - $SCHRODINGER/run scale_xy_z.py <input> <output> <scaling factor for x and y> <scaling factor for z>
    - (example command for the ALC-0315 system) $SCHRODINGER/run scale_xy_z.py md_prep_1/ALC-0315-run1_system-out.cms ALC-0315-run1_system_xy_55.cms 0.647 1.0
  - Copy (or move) the scaled cms file to "run_1us" directory and go to that directory (command line)
- Recommended procedure for resolving ring spears (if ring spears are found)
  - select the 'spear' residue within the two complexed
  - using 'Move' within the build toolbar, move the residue laterally a couple angstroms, until the structures are no longer clipping
  - select both the 'spear' residue that was just moved as well as the 'ring' residue
  - minimize both the residues quickly, and repeat for the next spear
  - repeat for rest of ring/spears, until when 'locate rings and spears' lists no ring/spears

**Step 3: Run 1μs MD simulation** (directory: run_1us)

- Goal: Relax the system
- Tasks
  - Edit the run_1us.sh to adjust -HOST, input and output cms and run the script to start the simulation (copy the files as needed to the machine you will use).

**Step 4: Umbrella sampling** (directory: umbrella_sampling)

- Goal: prepare files (6 replicas) and run umbrella sampling jobs (“relax” and “sampling”) for both of positively charged and neutral ionizable lipids
- Tasks
    - Import the output cms from Step 3 (run_1us-out.cms) into Maestro
    - Load the trajectory and go to the last frame
    - Select 6 ILP and 6 ILN molecules with a variety of Z positions of the Nitrogen atom
        - To make the operation easier:
            - Hide the water
            - Use CPK rendering for the Nitrogen atom of ILP or ILN
            - Put atom labels on selected atoms: XYZ coordinate, Atom number by entry
        - Center the structure on DSPC (res.ptype DSPC). This will ensure that Z=0 is in the middle of the bilayer.
        - Select 6 ILPs and 6 ILNs from the layer in the **positive** Z direction.
        - Note the XYZ coordinates of the N atom of the selected lipid, and select an atom (any atom) that is at the mirror image of the N atom.
            - For example, XYZ of N = (10.03 22.03 27.46)
            - Look for an atom (any atom) that is near (10.03 22.03 -27.46) (< +/- 1 would be nice. <+/- 1.5 is acceptable.)
        - Select these two atoms above. They are at the mirror image positions across Z, but they could be located in any position in XY. Make them the center of the XY plane by clicking “Use Workspace Selection” (with these two atoms selected) in the playback settings of the trajectory viewer.
        - Export the structure (“Current frame only”) to the Project Table. Edit the Title of this entry to make it informative. For example, ALC-0315 P34197(protonated, atom number of N=34197) or ALC-0315 N37494 (neutral, atom number of N=37494)
        - For each of the 12 structures (6 ILP and 6 ILN), run Prepare for MD (toggle ON for “Redistribute heavy atom mass to hydrogens”), and save the output cms files.
    - Place the 12 cms files (output of prepare MD from the previous step) in the “umbrella_sampling” directory
    - Create a folder “replica*n*” for each one of the six replicas
    - Copy one ILPcms file to replica1, another one to replica2, the third one to replica3, the fourth one to replica4, the fifth one to replica5, the sixth one to replica6. Also copy one ILN cms file to replica1, another one to replica2, etc. Each replica directory should have a pair of cms files (it does not matter how they are paired).
        - *Example: ALC-0315_P47057.cms (positively charged, atom number of N=47057) and ALC-0315_N41679.cms (neutral, atom number of N=41679).*

- Edit “build_umbrella.json” to specify the location of your “scripts” directory (on the machine you will run jobs)
- Copy build_umbrella.json to each replica*n* directory
- Edit “build_umbrella.json” in each directory to have the correct ILP and ILN file name and numbers
- From each replica directory, run “build_umbrella_full.py”
  - $SCHRODINGER/run ../build_umbrella_full.py
  - This will create “neutral” and “positive” directories, as well as “relax” and “sampling” sub-directories containing input files for umbrella sampling
- Run “relax” jobs (12 jobs, 2 per each replica)
  - Copy the entire umbrella_sampling directory to the machine you will run jobs
  - Each “relax” directory contains a .sh file with the commands
  - Confirm that SCHRODINGER_UMBRELLA_SAMPLING_1D is set to your scripts directory (on the machine you will run jobs) and edit the -HOST setting

- Confirm that a “relax” job is completed
  - The log will state “Calculation complete”
  - There should be 57 *_relax_no_mobile_restraints-out.cms files created

    ls *_relax_no_mobile_restraints-out.cms | egrep -c no_mobile → 57

- Go to the corresponding “sampling” directory (cd ../sampling)
  - Move all *_relax_no_mobile_restraints-out.cms files here

    mv ../relax/*_relax_no_mobile_restraints-out.cms .
  - Run the .sh script in this directory

- Upon completion of sampling jobs, 12 .fes files and 12 .pdf files (6 from ILP and 6 from ILN) will be created.
